# Rethinking EPO: A Paradigm Shift in Oncology?

**DOI:** 10.3390/cancers17233875

**Published:** 2025-12-03

**Authors:** Jean-Marc Ferrero, Baharia Mograbi, Rym Bouriga, Jocelyn Gal, Gérard Milano

**Affiliations:** 1 Department of Medical Oncology, Antoine Lacassagne Center, University Côte d’Azur, 33 Avenue de Valombrose, 06189 Nice, France; jean-marc.ferrero@nice.unicancer.fr; 2 Institute for Research on Ageing and Cancer, Nice (IRCAN), Institut Hospitalo-Universitaire (IHU) RespirERA, Fédérations Hospitalo-Universitaires (FHU) OncoAge, Centre National de la Recherche Scientifique (CNRS) 7284, Institut National de la Santé Et de la Recherche Médicale (INSERM) U1081, University Côte d’Azur, 06107 Nice, France; baharia.mograbi@univ-cotedazur.fr; 3 Medical Oncologist, Park Imperial Clinic, 28 Boulevard du Tzarewitch, 06000 Nice, France; dr.bouriga.cpi@sedna-sante.com; 4 Department of Epidemiology and Biostatistics, Antoine Lacassagne Center, University Côte d’Azur, 33 Avenue de Valombrose, 06189 Nice, France; jocelyn.gal@nice.unicancer.fr; 5 Scientific Valorization, Antoine Lacassagne Center, University Côte d’Azur, 33 Avenue de Valombrose, 06189 Nice, France

**Keywords:** erythropoietin, cancer treatment, anemia, immunotherapy, macrophage

## Abstract

Erythropoietin (EPO) is a hormone mainly produced by the kidney. EPO stimulates red blood cell production in response to hypoxia. EPO represents one of the most compelling paradoxes in medical oncology. For decades, recombinant EPO has been prescribed as an effective therapy for cancer-related anemia and fatigue, significantly improving patients’ quality of life while reducing their dependence on blood transfusions. Yet, accumulating scientific evidence knowledge has highlighted the complex interactions between EPO and tumor biology that extend well beyond its hematopoietic functions. The putative expression of EPO receptors on tumor cells has raised concerns about a potential role of EPO in promoting tumor progression, although conclusive clinical evidence remains lacking. Recent studies have revealed that EPO may contribute to immunosuppressive mechanisms within the tumor microenvironment, thereby reshaping our understanding of its risk/benefit profile in oncology. This evolving body of evidence calls for an objective reassessment of EPO’s dual nature as both a therapeutic ally and a potential oncological threat, in order to bring more caution around decision-making in the current era of strongly evolving cancer care.

## Simple Summary

Erythropoietin (EPO) is a hormone produced by the kidney that stimulates red blood cell production in response to hypoxia. EPO may contribute to immunosuppression within the tumor microenvironment. In addition, the presence of EPO receptors on tumor cells may promote tumor progression use in cancer care. The understanding of the benefit–risk balance of recombinant-EPO (r-EPO) has deepened, and greater caution is now required before initiating r-EPO treatment in cancer patients.

## 1. Background

### Erythropoietin (EPO): Mechanism of Action and Use

Erythropoietin (EPO) is a glycoprotein with a molecular weight of 30,400 Da that stimulates the production of erythrocytes and helps maintain their viability [1,2]. In the foetus, EPO is produced by the hepatocytes, while in adults, it is produced by interstitial peritubular renal cells. EPO plasma levels are variable and typically range from 4 to 26 mU/mL. The biosynthesis of EPO is regulated by several factors, including tissue hypoxia, a low red blood cell count, a reduction in oxygen pressure, and basically any condition that decreases the amount of oxygen delivered to tissues. At the cellular level, the synthesis of EPO is controlled by the transcription rate of the EPO gene [3]. This process is controlled by hypoxia-inducible factors (HIFs), particularly the HIF-2α subunit, which is highly sensitive to oxygen levels [4]. Briefly, tumor hypoxia stabilizes HIFs, primarily HIF-1α and HIF-2α. While HIF-1α is involved in metabolic changes and blood vessel formation, HIF-2α plays a crucial role in red blood cell production by activating the EPO gene [5]. Under normal oxygen conditions (normoxia), HIF-α subunits are continuously produced, hydroxylated, recognized by the von Hippel–Lindau (VHL) E3 ubiquitin ligase, and are rapidly degraded via the proteasome [6]. When hypoxia occurs, this hydroxylation is halted, preventing degradation and allowing HIF-2α to accumulate. Once stabilized, HIF-2α translocates to the nucleus, dimerizes with HIF-β, and binds hypoxia-response elements (HREs) in the EPO gene promoter to drive EPO transcription. Thus, EPO production exemplifies the broader HIF-regulated adaptive program that enables cells to survive and function under oxygen-limiting conditions.

**Figure 1:**
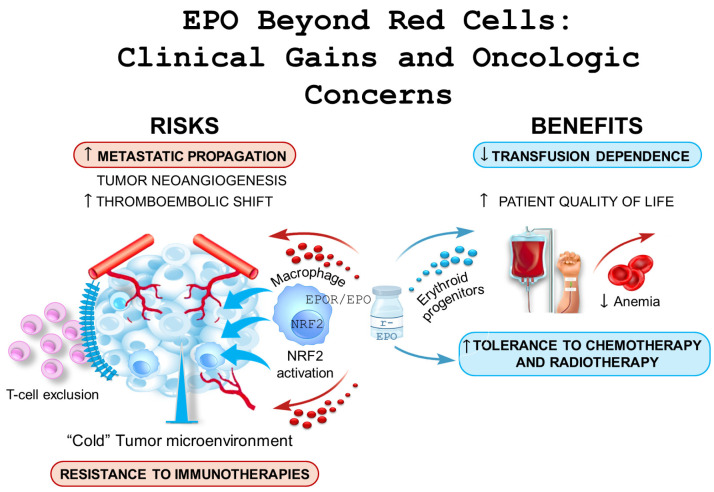
A summary of risks and benefits linked to the prescription of EPO-related compounds.

EPO exerts its cellular effects through the presence of EPO receptor molecules (EPO-R), as seen in Figure 1. When EPO binds to its receptor, it triggers an intracellular cascade of kinases that ultimately activates the transcription of proteins with anti-apoptotic functions. This process promotes differentiation and proliferation in various tissues, depending on where the EPO/EPO-R interaction occurs [7]. More precisely, the human EPO-R is primarily expressed in erythropoietin progenitors, but it is also found in several other tissues, including endothelial, cardiac, renal, and neuronal cells [8].

Anemia in cancer can arise from various factors, particularly from the infiltration of tumor cells into the bone marrow [9]. Chemotherapy can also have detrimental effects on blood cell precursors [10]. Specific agents, like cisplatin, can affect the renal tubules where EPO is synthesized [11]. Recombinant EPO (r-EPO) primarily serves to correct anemia [12]. Numerous clinical trials demonstrated the efficacy of r-EPO in managing cancer-related anemia and in preventing and correcting anemia resulting from cytotoxic treatments [13,14]. As a significant additional benefit of r-EPO administration in cancer patients, there is the well-characterized clinical capacity to reduce fatigue [15]. The primary objective of r-EPO treatment remains to maintain hemoglobin levels above the transfusion threshold, weighed against the risks and benefits of its administration [16]. Current recommendations state that erythropoiesis-stimulating agents including r-EPO treatments (such as epoetins or darbepoetin alfa) can be initiated when hemoglobin levels fall below 10 g/dL [3]. However, there is uncertainty regarding the potential risks associated with EPO derivatives in cancer patients. Several guidelines have been developed (Table 1), with a practical recommendation that the target hemoglobin concentration following r-EPO treatment should be strictly maintained between 12 and 13 g/dL [17]. In greater detail, Table 1 summarizes the different guidelines on r-EPO prescription and attempts to distinguish therapeutic contexts globally associated with either risks or benefits.

Here, we aimed to highlight some biological relevant aspects regarding EPO and EPO-R. We conducted a PubMed search using the keywords EPO, r-EPO, reviews, and meta-analyses, giving priority to studies with the largest patient populations and the most recent publications. Our attention was focused on epoetin and darbepoetin, excluding pegylated EPO [23], as this latter form is synthetic EPO that does not strictly match the native biological form of EPO considered in this review.

We also reviewed the safety concerns related to r-EPO use in cancer care, with particular attention to the ongoing debate about its potential negative impact on overall patient survival. Importantly, new light has been shed on this issue with the demonstration that the EPO/EPO-R axis acts as an immunosuppressive mechanism switch orchestrated by macrophages, Figure 1. This mechanism has been shown to be responsible for creating a tumor microenvironment that lacks T cells, thereby playing a crucial role in disrupting the homeostasis of antitumor immunity [24].

## 2. EPO and the Risk of Cancer Progression

### 2.1. The Presence of EPO-R on Tumors

There is cumulative evidence that r-EPO, as well as endogenous EPO, bind to and activate EPO-R not only on erythrocyte progenitors in the bone marrow but also on tumor cells [25]. Particularly challenging in this context is a retrospective, biological analysis of tumor samples from head and neck cancer patients showing that variable expression of EPO-R on tumor cells could be associated with a detrimental clinical outcome in patients treated with r-EPO [26]. In contrast, a review by Aapro and coworkers [27] critically analyzed the evidence regarding the presence of EPO-R in tumor tissues and cell lines. The review highlighted that technical issues may have compromised the validity of previous findings concerning the presence of EPO-R in tumors. More precisely, the studies cited used Western blotting and immunohistochemistry, which may have produced false-positive results due to the lack of specificity of commercially available polyclonal or monoclonal anti-EPO-R antibodies [28]. The use of an optimized monoclonal antibody with improved specificity led subsequent studies to indicate that many tumor cell lines express EPO-R at potentially low or even undetectable levels [29]. Moreover, LaMontagne and colleagues [30] questioned the negative role of EPO-R presence in tumors exposed to EPO. Their experimental investigations conducted on breast carcinoma models were unable to demonstrate a positive link between EPO-R expression and the stimulating effects of r-EPO on tumor growth and migration. In contrast, we, and other researchers, have reported that the expression of EPO-R in CAL-166 head and neck cancer cells did not appear to proportionately influence their growth [31].

### 2.2. EPO and Tumor Microenvironment

A pro-angiogenic ability of EPO sustains an interaction between EPO and the tumor micro-environment (TME). Kimakova and colleagues reviewed the impact of EPO on vascular endothelial cells [32]. EPO appears to activate signaling pathways in endothelial cells and influences target genes associated with neoangiogenesis. One of the key components regulated through EPO signaling is vascular endothelial growth factor (VEGF), which acts as a growth factor for endothelial cells within the tumor microenvironment [33]. Under pathological conditions, particularly in response to hypoxia, often associated with tumor progression, elevated VEGF levels promote the formation of abnormal blood vessels in the tumor microenvironment. Notably, VEGF and EPO synergistically cooperate in the process of vascularization [34]. This finding may be explained by EPO’s ability to induce the expression of VEGF receptors [35]. Tumorigenic effects of EPO, independent of any direct impact on tumor cells, have also been reported. For instance, EPO has been shown to accelerate the growth of rat tumor xenografts that lack EPO-R, by enhancing angiogenesis in vivo [36]. Collectively, these findings suggest caution regarding the potential detrimental effects of r-EPO on tumor growth through favored neovascularization processes.

### 2.3. r-EPO Use and Disease Progression

There is currently no clear consensus regarding the potential risk of tumor progression associated with EPO-R activation, as pooled results from meta-analyses have yielded divergent conclusions [37]. More recent meta-analyses on the existence [38] or not [39] of a risk of unfavorable cancer evolution under r-EPO underline this climate of doubt. Strengthening this clinical uncertainty, the univocal biological mechanisms explaining the possible detrimental effects of r-EPO on cancer progression have still not been completely elucidated [40]. The absence of a clear-cut conclusion from the meta-analyses compiled by Dicato and coworkers [37] may also be attributable to recurrent methodological biases across the meta-analyses considered. In particular, it is known that meta-analyses may amplify biases, propagate heterogeneity, and mask true clinical effects when flawed or heterogeneous trials predominate [41,42].

### 2.4. EPO and Cancer Immunity

A recent, provocative, experimental study published by Chiu and colleagues [24] stands out in the overall, blurred context of EPO and cancer progression risk. The authors conducted a large set of in vitro and in vivo experimental investigations, making an important contribution to the EPO-cancer field; they suggested that EPO can promote an immunosuppressive, non-inflamed tumor microenvironment (TME) [24]. Non-inflamed tumors are refractory to immunotherapy, particularly in the case of checkpoint inhibitors (CPIs) [43]. CPIs refractory tumors are typically populated by immunosuppressive macrophages and neutrophils, which create barriers that prevent cytotoxic T cells from accessing the tumor and significantly hamper the development of an effective antitumor immunity. Chiu and coworkers focused on spontaneous preclinical models of hepatocarcinoma with distinct immunotypes and demonstrated that an activated EPO/EPO-R pathway can induce immunosuppression by binding to EPO-R on tumor–associated macrophages. This interaction prevents their differentiation into immune-stimulatory macrophages. At a mechanistic level, the authors identified the transcription factor NRF2 (nuclear factor erythroid 2-related factor 2) as a central downstream mediator of the EPO/EPO-R interaction, controlling antioxidant production in macrophages and potentially driving their immunological reprogramming. This mechanism resulted globally in a cytotoxic T cell-deprived TME, thus constituting a poor environment for an effective antitumor immunity [24]. The authors concluded that the EPO/EPO-R-driven axis could represent a valuable therapeutic target in hepatocarcinoma. However, in our opinion, there is an even more significant consequence of the data obtained that draws attention to the potential threat associated with EPO exposure (whether local or systemic) by creating an immunosuppressive tumor environment. Globally, these findings bring an important contribution to the still open questioning regarding the multifaceted aspects relative to the EPO use in cancer therapy.

Additionally, the study by Chiu and coworkers and immediate comments on it [44] suggest a potential influence of r-EPO intake on the optimal efficacy of immunotherapy by CPIs. The debate surrounding the optimal predictive factors for the application of CPIs remains open, especially when facing the increasing trend to develop combinations of immunotherapy and chemotherapy, the latter of which potentially triggering a need for supplemental recombinant EPO. Consequently, retrospective analyses are encouraged to investigate whether r-EPO intake could negatively affect or not outcomes during the course of immunotherapy. In this respect, considering EPO circulating levels as part of a multifactorial approach to predict CPI treatment outcome should be particularly relevant. Applying artificial intelligence algorithms could offer a promising avenue to conduct the appropriate data-rich multifactorial studies [45].

## 3. Conclusions and Perspectives

The EPO story in cancer highlights a therapeutic landscape marked by both promise and questioning. While EPO continues to offer invaluable relief from cancer-related anemia, its ability to promote tumor progression and immunosuppressive microenvironments cannot be neglected. Recent evidence suggesting that EPO may compromise the efficacy of checkpoint inhibitor immunotherapy may complicate clinical decision-making, particularly as combination therapies become increasingly prevalent in modern oncology. Moving forward, the oncology community should exercise a greater vigilance in carefully weighing the benefits and risks of r-EPO for cancer patients (Figure 1). These new findings also emphasize the need for continued research into safer alternatives and optimized treatment protocols in our ongoing fight against cancer, where an optimal use of approved drugs is still under debate [46].

Since r-EPO may potentially have a harmful effect on tumor progression, it is clear that the risk of accelerated progression due to r-EPO treatment remains under debate, and objective evidence is still awaited to formulate a definitive answer [2,47]. Based on the provocative study by Chiu and coworkers [24], additional investigations should be conducted to more deeply explore confirm the potential immunosuppressive effect of EPO, which introduces a new layer of complexity concerning this growth factor. Among the various options for supplementary, potentially confirmatory studies, it is important to consider both a wide range of EPO-R-expressing tumors and to clarify the exact molecular mechanisms underlying the EPO-induced shift in macrophage immune status. The proposed impact of EPO on NRF2 activation needs to be validated, and other, more specific potential pathways should be explored, as NRF2 regulates the expression of a particularly large number of genes involved in intracellular redox balance and inflammation [48]. Advances in the fast–developing field of single-cell omics technologies combined with computational strategies will certainly provide more extended information on the cellular impact of EPO following its initial cellular interaction with EPO-R [49].

Furthermore, since EPO-R is produced in various immune cells, including T lymphocytes [50], EPO could potentially modulate their activation and functions, thereby extending its immunosuppressive effects beyond macrophages in the TME-immune cell interaction.

Overall, one can estimate that the notion of benefit/risk inherent in r-EPO use has now deepened and more caution should be present in mind for patients to be treated with r-EPO.

## Figures and Tables

**Table 1:** r-EPO use and recommendations overview in cancer patients.

r-EPO Recommended	r-EPO Not Recommended	Arguments and Strength of Evidence		References
**Patients with chemotherapy-induced anaemia, especially when treatment is non-curative, for instance when transfusion avoidance is desirable (e.g., limited blood supply, alloimmunization risk)**		r-EPO increases hemoglobin levels and reduces RBC transfusion needs but may worsen survival in curative settings.	**High** Multiple RCTs and meta-analyses; Grade A (ASCO/ASH)	[18]
	**Patients not receiving chemotherapy** (no myelosuppressive therapy)	r-EPO is not recommended for cancer patients not on chemotherapy, except in rare palliative cases.	**High** Evidence-based clinical practice guideline	[19]
**Patients in non-curative-intent settings**		The ASCO/ASH guidelines support the use of r-EPO in non-curative chemotherapy settings.	**High** Cochrane meta-analysis (60+ RCTs)	[20,21]
	**Patients in** **curative-intent settings**	r-EPO increases hemoglobin levels and reduces RBC transfusion needs but may worsen survival in curative settings.	**High** Multiple RCTs and meta-analyses; Grade A (ASCO/ASH)	[18]
**Patients without major thrombotic risk factors**	**Patients with high risk of thromboembolic events** (history of thrombosis, predisposition)	The ASCO/ASH guidelines support r-EPO increases risk of VTE (OR ≈ 1.48).	**High** Cochrane meta-analysis (60+ RCTs)	[20,21]
	**Aggressive tumour types** (head and neck radiotherapy, cervical chemoradiotherapy, metastatic breast, lung cancer)	Some trials observed worse outcomes when r-EPO were used with aggressive pro-thrombotic tumor types where endothelial damage is amplified by radiotherapy or high Hb targets (>12 g/dL).	**Moderate** meta-analyse (lung cancer subgroup)	[22]

Keys: RCT = Randomized controlled trial; VTE = Venous Thromboembolism; OR = Objective risk; Grade A = High-quality evidence.
